# The impact of systemic precision medicine and immunotherapy treatments on brain metastases

**DOI:** 10.18632/oncotarget.27328

**Published:** 2019-11-19

**Authors:** Rowland H. Han, Gavin P. Dunn, Milan G. Chheda, Albert H. Kim

**Affiliations:** ^1^ Department of Neurological Surgery, Washington University School of Medicine, St. Louis, MO, USA; ^2^ Center for Human Immunology and Immunotherapy Programs, Washington University School of Medicine, St. Louis, MO, USA; ^3^ Department of Medicine, Washington University School of Medicine, St. Louis, MO, USA; ^4^ Department of Neurology, Washington University School of Medicine, St. Louis, MO, USA

**Keywords:** brain metastases, targeted therapy, precision medicine, immunotherapy, systemic therapy

## Abstract

Metastases from melanoma, lung and breast cancer are among the most common causes of intracranial malignancy. Standard of care for brain metastases include a combination of surgical resection, stereotactic radiosurgery, and whole-brain radiation. However, evidence continues to accumulate regarding the efficacy of molecularly-targeted systemic treatments and immunotherapy. For non-small cell lung cancer (NSCLC), numerous clinical trials have demonstrated intracranial activity for inhibitors of EGFR and ALK. Patients with melanoma brain metastases may benefit from systemic therapy using BRAF-inhibitors with and without trametinib. Several targeted options are available for breast cancer brain metastases that overexpress HER2, although agents with intracranial activity are still needed for other molecular subtypes. Immune checkpoint inhibitors including anti-CTLA-4 and anti-PD-1/PD-L1 antibodies are yielding impressive responses in intracranial manifestations of metastatic melanoma and NSCLC. Given the promising early results with these emerging therapies, management of eligible patients will require increased multidisciplinary discussion incorporating novel systemic treatment approaches prior or in addition to local therapy.

## INTRODUCTION

Metastases from systemic cancers are the most common type of intracranial malignancy, and the most common cancers that lead to brain metastases (BM) are lung (16.3% 5-year cumulative incidence), renal (9.8%), melanoma (7.4%), and breast (5.0%) [1]. The incidence of BM is likely increasing because patients are living longer due to more effective systemic therapies, as well as increasing use of screening neuroimaging. Traditionally, standard therapy for BM has focused on local treatment including craniotomy for resection and/or stereotactic radiosurgery (SRS), with whole-brain radiation therapy (WBRT) reserved for more disseminated intracranial disease [2]. A recent set of guidelines produced by the Congress of Neurological Surgeons (CNS) and the American Association of Neurological Surgeons/CNS Section on Tumors provides updated recommendations regarding the roles of surgery, SRS, and WBRT but concluded that there is insufficient evidence to make recommendations regarding molecularly targeted agents [3, 4].

**Figure 1:**
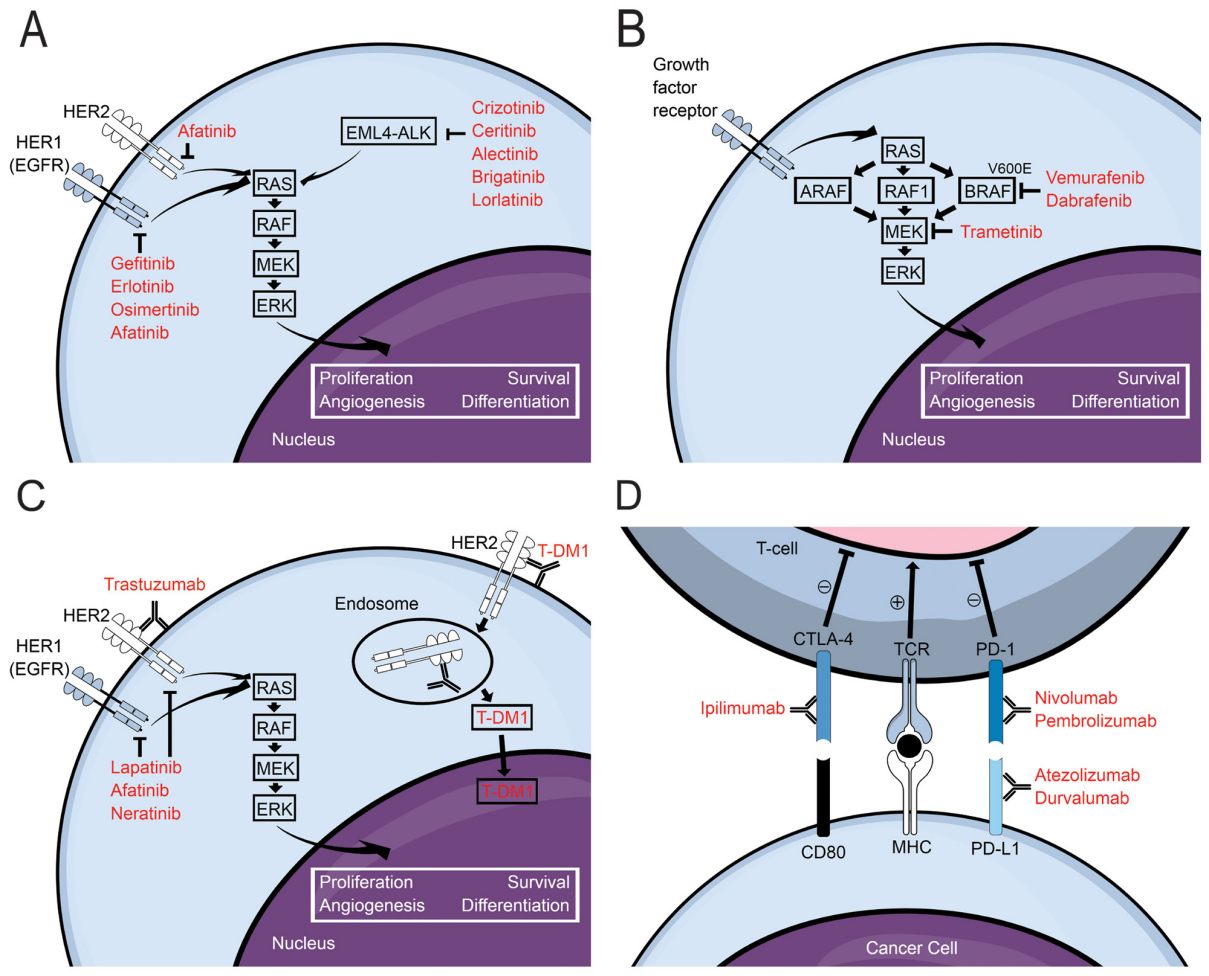
Schematic diagrams illustrating mechanisms of action for **(A)** EGFR- and ALK- inhibitors for non-small cell lung cancer, **(B)** BRAF- and MEK- inhibitors for metastatic melanoma, **(C)** HER2-targeted therapies for breast cancer, and **(D)** immune checkpoint inhibitors. For additional information regarding PD-L1 inhibitors please see article by O’Kane and Leighl [83]. Abbreviations: HER: human epidermal growth factor receptor; EGFR: epidermal growth factor receptor; EML4: echinoderm microtubule-associated protein-like 4; ALK: anaplastic lymphoma kinase; T-DM1: trastuzumab emtansine; CTLA-4: cytotoxic T-lymphocyte-associated protein 4; CD80: cluster of differentiation 80; TCR: T-cell receptor; MHC: major histocompatibility complex; PD-1: programmed cell death protein 1; PD-L1: programmed death-ligand 1.

Although attractive in many cases, the utility of conventional systemic therapy in the treatment of BM has historically been limited due to poor penetration across the blood-brain (BBB) and blood-tumor barrier [5]. Traditional clinical trials for systemic therapies have excluded patients with symptomatic or uncontrolled BM due to these challenges with central nervous system (CNS) penetration [1]. More recently, targeted systemic therapies have demonstrated improved extracranial disease control and survival in molecularly defined subpopulations, and the ability of these medications to complement or even replace local treatment of BM is under intense investigation [6]. In fact, a series of recent studies have importantly suggested that many targeted agents are able to achieve therapeutic concentrations in the brain, including EGFR- and ALK- inhibitors for non-small cell lung cancer, BRAF- and MEK- inhibitors for melanoma, and HER2-directed therapies for breast cancer. In addition, systemically delivered immune therapies such as checkpoint blockade have also demonstrated efficacy in the CNS [7]. Furthermore, while earlier trials were difficult to interpret due to variations in response and progression criteria, the Response Assessment in Neuro-Oncology Brain Metastases (RANO-BM) provided standardization in study assessments [8]. This review summarizes emerging evidence for targeted systemic therapies for the most common sources of BM, which are illustrated in Figure 1.

## LUNG CANCER BRAIN METASTASES

Lung cancer is the leading cause of cancer death overall, and 13-44% of patients with lung cancer ultimately develop BM [9–11]. Although BM are more common in small cell lung cancer, most studies have focused on the targeted treatment of metastases from non-small cell lung cancer (NSCLC) because the latter represents a greater proportion (80-85%) of total lung cancer cases [9]. NSCLC comprises a heterogeneous group of cancers including squamous cell carcinoma, large cell carcinoma, and adenocarcinoma. Several molecular driver mutations have been identified within NSCLC including mutation of epidermal growth factor receptor (EGFR), as well as fusion of anaplastic lymphoma kinase (ALK) and echinoderm microtubule-associated protein-like 4 (EML4) genes [1]. Targeted therapies for NSCLC BM have focused on inhibitors of these subclasses of tumors (Table 1).

**Table 1:** Selected clinical studies of targeted treatments for non-small cell lung cancer brain metastases

Authors & Year	Regimen	Target (gen.)	*N*	cRR (%)	PFS (mo)	OS (mo)
Kim et al., 2009	Gefitinib or erlotinib	EGFR (I)	23	73.9	7.1	18.8
Iuchi et al., 2013	Gefitinib	EGFR (I)	41	87.8	14.5	21.9
Welsh et al., 2013	Erlotinib + WBRT	EGFR (I)	40	86	8.0	11.8
Hoffknecht et al., 2015	Afatinib	EGFR (II)	100	35	3.6	9.8
Kim et al., 2016 (ASCEND-1)	Ceritinib	ALK (II)	94	68	--	--
Crino et al., 2016 (ASCEND-2)	Ceritinib	ALK (II)	100	45	5.4	--
Soria et al., 2017 (ASCEND-4)	Ceritinib	ALK (II)	121	46.3	10.7	--
Shaw et al., 2017 (ASCEND-5)	Ceritinib	ALK (II)	66	35	4.4	--
Gadgeel et al., 2014	Alectinib	ALK (II)	21	52	--	--
Gadgeel et al., 2016	Alectinib	ALK (II)	136	42.6	11.1	--
Novello et al., 2018 (ALUR)	Alectinib	ALK (II)	40	54.2	--	--
Camidge et al, 2018 (ALTA)	Brigatinib	ALK (II)	59	46-67	14.6-18.4	--
Shaw et al., 2017	Lorlatinib	ALK/ROS1 (III)	24	46	--	--
Solomon et al., 2018	Lorlatinib	ALK/ROS1 (III)	165	53.1-87.0	--	--

Abbreviations: cRR: CNS response rate; gen: generation; mo: months; *N*: # patients; OS: overall survival; PFS: progression free survival; WBRT: whole-brain radiation therapy.

### EGFR inhibitors

Mutation of EGFR increases its kinase activity thereby promoting tumor cell survival in 10-35% of NSCLC [12]. First generation EGFR inhibitors bind reversibly to the tyrosine kinase of EGFR, whereas second-generation agents bind irreversibly and are therefore more potent. Third generation EGFR inhibitors have less adverse effects and are active against tumors with acquired EGFR^T790M^ mutations [8]. The two first-generation EGFR inhibitors, erlotinib and gefitinib, have shown potent activity within the CNS. However, most prospective studies evaluating the efficacy of erlotinib or gefitinib for NSCLC BM have used them as second-line agents in patients who had been previously treated with radiotherapy or chemotherapy [13, 14].

Two published phase II trials have evaluated first-generation EGFR inhibitors (gefitinib or erlotinib) as first-line therapy in patients with EGFR-mutant NSCLC with BM [15, 16]. None of the patients in these trials had received prior treatment with chemotherapy or radiotherapy. Both studies showed favorable response of BM to either gefitinib or erlotinib with progression free survival (PFS) between 7.1 and 14.5 months and overall survival (OS) between 18.8 and 21.9 months. Additional studies have evaluated the efficacy of radiotherapy combined with EGFR inhibitors in radiotherapy-naïve patients with EGFR-mutated NSCLC BM [17–19]. One clinical trial using erlotinib concurrently with WBRT showed improved PFS (12.3 versus 5.2 months) and OS (19.1 versus 9.3 months) in patients with EGFR mutations compared to those without [20]. Intracranial response rate within the study cohort was 86%, including 31% who experienced complete response. Overall, the first-generation EGFR inhibitors, gefitinib and erlotinib, appear to be effective in controlling BM both with and without concurrent radiation in patients with EGFR-mutant NSCLC.

At present, data are sparse regarding the second and third-generation EGFR inhibitors, afatinib and osimertinib, respectively, in treatment of NSCLC BM. One prospective study assessed the efficacy of afatinib in 100 patients with NSCLC BM [21]. These patients had all failed at least one prior platinum-based chemotherapy as well as a first-generation EGFR inhibitor. PFS in patients treated with afatinib was 3.6 months, and cerebral response rate was 35%. Further clinical trials on the efficacy of afatinib for the treatment of NSCLC BM are underway (NCT02768337). The third-generation EGFR inhibitor, osimertinib, has been approved for patients with a specific EGFR mutation (T790M) who have failed prior tyrosine kinase inhibitor treatment [22]. Preclinical studies have shown good CNS penetration and activity of third-generation EGFR inhibitors [23], and additional clinical trials are underway (NCT02972333, NCT02736513, NCT02971501). The efficacy of second and third-generation EGFR inhibitors for the management of NSCLC BM requires further study before they can be recommended for clinical use.

### ALK inhibitors

ALK-positive NSCLC make up approximately 5% of patients with NSCLC metastases and are characterized by a fusion of ALK and EML4 genes. These patients are of particular interest because they comprise primarily younger patients with little or no smoking history and for which EGFR inhibitors do not work [24, 25]. BM appear to be more common in patients with ALK-positive NSCLC compared to unselected NSCLC [9, 26]. Several notable ALK inhibitors have been studied with positive effects in ALK-positive NSCLC BM [27].

The first-generation ALK inhibitor, crizotinib, was approved in 2011 for treatment of patients with locally advanced or metastatic ALK-positive NSCLC. A retrospective analysis of two randomized clinical trials (PROFILE 1005 and 1007) examined the intracranial response to crizotinib in patients with ALK-positive NSCLC BM, and found intracranial disease control rates of 56-62% [28]. However, several studies have found CNS disease progression resulting from acquired resistance to crizotinib [29–31].

Subsequently, two second-generation ALK inhibitors, ceritinib and alectinib, were found to have improved CNS penetration and are now approved for the treatment of ALK-positive metastatic NSCLC in patients who previously failed crizotinib [9]. An open-label phase I trial assessed the safety of ceritinib in 246 patients (ASCEND-1), and efficacy was determined in *post-hoc* analysis [32]. In this trial, 94 (38%) patients had confirmed BM and follow-up neuroimaging. Intracranial disease control with ceritinib was 79% and 65% in ALK-inhibitor naïve and previously ALK-inhibitor treated patients, respectively. Intracranial activity of ceritinib has been confirmed in several follow-up phase II/III studies (ASCEND 2-5) [33–35]. An open-label, multicenter phase II trial is ongoing to assess the safety and efficacy of ceritinib in patients with ALK-positive NSCLC and brain or leptomeningeal metastases (NCT02336451). At present, ceritinib appears to be effective in controlling BM from ALK-positive NSCLC and may be more beneficial when used prior to crizotinib.

Following the phase I trial for alectinib in patients with ALK-positive NSCLC, a multi-center, single-group, open-label phase II trial was undertaken in North America [36, 37]. All 87 patients in this trial had baseline CNS imaging with MRI or CT, and 16 (18%) had measurable CNS disease at baseline. Of these, 11 (69%) had received prior brain radiation therapy. Complete CNS response was reported in 4 of the 16 patients, and partial response in an additional 8 of 16. Median duration of CNS response was 11.1 months. A global phase II trial assessing 138 patients with ALK-positive NSCLC who were treated with second-line alectinib after failing crizotinib showed similar results [38]. A pooled analysis of these two trials included 225 total patients, 136 (60%) of which had CNS metastases at baseline (50 measurable, 86 unmeasurable) [39]. All patients had been previously treated with crizotinib and 95 (70%) had already undergone radiation therapy. Complete CNS response was seen in 37 (27.2%) patients, partial response in 21 (15.4%), and 58 (42.6%) patients had stable CNS disease. Median CNS duration of response was 11.1 months.

Following the success of phase I and II trials for alectinib in ALK-positive NSCLC, several phase III studies focused on CNS disease [40–42]. The ALEX study included 122 patients with ALK-positive NSCLC and baseline BM who received either alectinib or crizonitib [43]. CNS response rate was 85.7% with alectinib versus 71.4% with crizonitib in patients with prior radiotherapy and 78.6% versus 40.0%, respectively, in those without prior radiotherapy. The ALUR study randomized a total of 107 patients with advanced ALK-positive NSCLC who were previously treated with crizotinib to receive either alectinib or chemotherapy [40]. Out of the 40 patients with baseline measurable CNS disease (24 alectinib, 16 chemotherapy), CNS response rate was higher with alectinib (54.2%) versus chemotherapy (0%). Together, these studies suggest robust response of ALK-positive NSCLC BM to alectinib both as initial and secondary ALK inhibitor therapy.

Another second-generation ALK-inhibitor, brigatinib, has shown promising intracranial disease activity in clinical trials [44, 45]. ALTA was a randomized phase II trial in which patients with ALK-positive NSCLC with baseline BM received varying doses of brigatinib [44]. Intracranial response rate among patients with measurable BM was 46-67% (total 59 patients). Median intracranial PFS was 14.6 to 18.4 months. Another open-label, randomized, phase III trial enrolled 275 patients with advanced ALK-positive NSCLC who were ALK-inhibitor naïve to receive brigatinib or crizotinib [45]. Among 39 patients with measurable brain lesions, intracranial response rate was 14 out of 18 (78%) with brigatinib versus 6 out of 21 (29%) with crizotinib. Therefore, brigatinib has improved intracranial activity compared to crizotinib and is efficacious in the treatment of ALK-positive NSCLC BM.

Finally, promising data are emerging regarding a third-generation dual-inhibitor of ALK and ROS proto-oncogene 1 (ROS1) with CNS penetrance, lorlatinib. An international multicenter, open-label phase I study enrolled 54 patients with advanced ALK-positive or ROS1-positive NSCLC to receive lorlatinib at varying doses, including 24 with baseline measurable BM [46]. Of these, 11 of 24 had intracranial objective response to the treatment drug (7 complete, 4 partial). This was followed by a phase II study which included 276 patients with ALK- or ROS1-positive NSCLC who underwent treatment with lorlatinib [47]. Study patients were divided into 6 cohorts on the basis of ALK and ROS1 status and previous therapy with crizotinib, other ALK-inhibitors, or chemotherapy. In patients with measurable baseline BM, objective intracranial responses were noted in 53.1-87.0% of patients with ALK-positive NSCLC. Lorlatinib is currently undergoing a phase III trial comparing its efficacy against crizotinib as first-line treatment for ALK-positive NSCLC (NCT02927340 and NCT03052608). Overall, lorlatinib demonstrates strong activity against ALK-positive NSCLC BM and may also be efficacious for ROS1-positive NSCLC.

## MELANOMA BRAIN METASTASES

The prevalence of BM in patients with malignant melanoma is as high as 50-75%, and survival of patients with multiple BM is generally 6 months or less [48–50]. Because patients with melanoma BM frequently have multiple small metastases, systemic targeted therapy is an attractive treatment option (Table 2) [9, 51, 52].

**Table 2:** Selected clinical studies of targeted treatments for melanoma brain metastases

Authors & Year	Regimen	Target	***N***	cRR (%)	PFS (mo)	OS (mo)
Dummer et al., 2014	Vemurafenib	BRAF	24	84	3.9	5.3
McArthur et al., 2017	Vemurafenib	BRAF	146	18	3.7-4.0	8.9-9.6
Falchook et al., 2012	Dabrafenib	BRAF	10	90	4.2	--
Long et al., 2012 (BREAK-MB)	Dabrafenib	BRAF^V600E^	139	30.8-39.2	16.1-16.6	31.4-33.1
Davies et al., 2017 (COMBI-MB)	Dabrafenib + trametinib	BRAF^V600E^/MEK	76	58	5.6	10.8

Abbreviations: cRR: CNS response rate; mo: months; *N*: # patients; OS: overall survival; PFS: progression free survival

### BRAF-inhibitors

The proto-oncogene BRAF is mutated in approximately 50% of malignant melanomas [53], and BRAF-mutant melanoma is associated with a higher rate of CNS involvement (24%) compared with BRAF wild type melanomas (12%) [54]. At present, two BRAF-inhibitors, vemurafenib and dabrafenib, are approved for the treatment of advanced malignant melanoma. An open-label phase II trial treated 24 patients with nonresectable, previously treated BRAF-mutated melanoma BM using vemurafenib [55]. In this cohort, median OS was 5.3 months and PFS was 3.9 months. Among 19 patients with measurable intracranial disease at baseline, 3 (16%) experienced partial response and 13 (68%) had stable intracranial disease. Another multicenter, open-label phase II trial examined the response of BM from BRAF-mutated melanoma to vemurafenib [56]. The study included 90 patients with previously untreated BM (cohort 1), and 56 patients who had received prior treatment for their BM (cohort 2). Overall intracranial response rate was 16 out of 90 (18%) in cohort 1, including two complete responses and 14 partial responses. Median OS and PFS were 8.9 and 3.7 months in cohort 1, respectively, and 9.6 and 4.0 months in cohort 2, respectively. Together, these studies suggest a modest response of intracranial disease to vemurafenib in BRAF-mutant melanoma.

Use of dabrafenib to treat melanoma BM has been explored in a phase I trial involving 184 patients who received the drug for incurable solid tumors [57]. Out of 10 patients who had asymptomatic, untreated BM, 9 had decrease in size of their BM, and 4 had complete resolution. Median PFS was 4.2 months in this subset of patients. A subsequent multicenter, open-label phase II trial included 172 patients with melanoma BM who were treated with dabrafenib [58]. Patients were divided by prior local treatment status and by type of BRAF-mutation (BRAF^V600E^ or BRAF^V600K^). Out of 74 patients with previously untreated BM and BRAF^V600E^ mutations, 29 (39.2%) had an overall intracranial response. Out of 65 patients who *had* undergone prior local treatment for BM and had BRAF^V600E^ mutations, 20 (30.8%) achieved overall intracranial response. OS and PFS were 33.1 and 16.1 months, respectively, for untreated patients, and 31.4 and 16.6 months, respectively, for previously treated patients. Response rates, OS, and PFS were lower in patients with BRAF^V600K^-mutant melanoma. Overall, dabrafenib has activity against BRAF^V600E^-mutant melanoma BM regardless of prior local treatment status.

### BRAF- and MEK-inhibitor combination therapy

One major challenge in treating metastatic melanoma with BRAF-inhibitors is the 13 development of resistance secondary to upregulation of other proteins in the Ras-Raf-MEK14 MAPK pathway, such as RAF1 (C-RAF) or RAS. This can lead to increased tumor cell 15 proliferation and even to the development of secondary skin tumors such as squamous cell 16 carcinomas [59–62]. This finding has led to trials combining treatment with BRAF-inhibitors and 17 the MEK-inhibitor, trametinib. An open-label phase III trial investigated the efficacy of 18 dabrafenib and trametinib combination treatment in 704 patients with BRAF-mutated metastatic 19 melanoma [63]. The study found that first-line combination dabrafenib-trametinib led to 20 improved 12 months OS (72% versus 65%) and longer median PFS (11.4 versus 7.3 months) 21 compared to vemurafenib monotherapy. This was followed by a multicenter, open-label, phase II 22 trial (COMBI-MB) that examined intracranial response to combination dabrafenib-trametinib in 23 patients with BRAF-mutant melanoma BM [64]. The study found intracranial response in 44 of 1376 (58%) patients, OS of 10.8 months, and PFS of 5.6 1 months in patients with BRAFV600E-2 mutant, asymptomatic BM with no prior local treatment. Overall, patients appear to benefit 3 clinically from dabrafenib-trametinib as evidenced by median OS of 10.1-24.3 versus 3.4 months 4 in historical controls treated with WBRT alone.

## BREAST CANCER BRAIN METASTASES

Breast cancer is the most common cancer in women, and 10-20% of breast cancer patients have BM [65, 66]. Breast cancers can be classified on the basis of estrogen receptor, progesterone receptor, and human epidermal growth factor receptor 2 (HER2) status. HER2 overexpression is present in approximately 25-30% of breast cancers, and these are 2-4 times more likely to result in BM [9]. Thus far, only HER2-positive breast cancers have found success in targeted therapy for BM (Table 3) [67, 68].

**Table 3:** Selected clinical studies of targeted treatments for HER2-positive breast cancer brain metastases

Authors & Year	Regimen	Target	***N***	cRR (%)	PFS (mo)	OS (mo)
Lin et al., 2009	Lapatinib +/- capecitabine	HER2/EGFR	242	6-20	2.4-3.7	6.4
Bachelot et al., 2013 (LANDSCAPE)	Lapatinib + capecitabine	HER2/EGFR	45	66	5.5	17
Freedman et al., 2019	Neratinib + capecitabine	HER2/EGFR	49	33-49	3.1-5.5	13.3-15.1
Krop et al., 2015 (EMILIA)	T-DM1	HER2	45	--	5.9	26.8
Bartsch et al., 2015	T-DM1	HER2	10	50	5	--

Abbreviations: cRR: CNS response rate; mo: months; *N*: # patients; OS: overall survival; PFS: progression free survival; T-DM1: trastuzumab emtansine.

Trastuzumab is a monoclonal humanized antibody directed against the extracellular domain of HER2 and is highly effective for the treatment of HER2-positive breast cancer [69–71]. Although trastuzumab cannot cross the intact BBB due to its large molecular size, studies have suggested trastuzumab penetration in BM secondary to BBB breakdown [72]. Retrospective studies have shown response of BM to intravenous trastuzumab [73], and prospective trials assessing the efficacy of trastuzumab in the treatment of HER2-positive breast cancer BM are underway (NCT02571530 and NCT01325207). Currently, however, evidence for the use of trastuzumab in the treatment of breast cancer BM is still accumulating.

Lapatinib is a small receptor tyrosine kinase inhibitor that interferes with both HER2 and EGFR signaling and is believed to cross the BBB, albeit to a limited extent in the commonly used dosing regimen [74]. An open-label, multicenter phase II trial included 242 patients with HER2-positive breast cancer and BM who had been treated with trastuzumab and a combination of WBRT and SRS, followed by lapatinib [75]. CNS partial response was seen in 6% of patients, stable disease in 37%, and progressive disease in 46%. There were no complete responses. OS in the study was 6.4 months and PFS was 2.4 months. An extension of the trial included 50 patients who received additional treatment with lapatinib plus capecitabine, resulting in CNS partial response rate of 20% (no complete responses) and PFS of 3.7 months. The LANDSCAPE trial was an open-label, multicenter phase II trial that evaluated the response of radiation-naïve HER2-positive breast cancer BM to first-line lapatinib-capecitabine [76]. Out of the total 45 patients, partial CNS responses were seen in 29 (66%), median OS was 17 months, and PFS was 5.5 months. Clinical trials investigating the efficacy of lapatinib in combination with WBRT or SRS for treatment of HER2-positive breast cancer BM are ongoing (NCT01622868). Two additional dual HER2 and EGFR inhibitors, afatinib and neratinib, are undergoing clinical trials for treatment of BM from HER2-positive breast cancer with promising results [77, 78]. In summary, dual HER2 and EGFR inhibitors exhibit modest CNS activity and may be used in the management of HER2-positive breast cancer BM.

Trastuzumab emtansine (T-DM1) is an antibody-drug conjugate of trastuzumab and the cytotoxic microtubule-inhibitor DM1 (also known as emtansine). This was approved for the treatment of metastatic HER2-positive breast cancer based on the phase III EMILIA trial which compared T-DM1 with lapatinib plus capecitabine and found improved PFS, OS, and objective response rate for T-DM1 [79]. A subsequent retrospective analysis of the EMILIA trial looked at the response rates of patients with BM at baseline and found significantly longer OS in those treated with T-DM1 (26.8 months) compared to lapatinib-capecitabine (12.9 months) [80]. A study examining T-DM1 as primary systemic therapy for BM from HER2-positive breast cancer found clinical benefit for CNS disease in 5 of 10 patients [81]. T-DM1 demonstrated CNS activity for HER2-positive breast cancer BM, but additional clinical studies are needed due to the small sample size and retrospective nature of existing evidence.

## IMMUNE CHECKPOINT INHIBITORS

Cancer immunotherapy refers to the modulation of the host’s immune system to treat malignancies and includes the use of checkpoint inhibitors (anti-CTLA-4 and anti-PD-1/PD-L1 antibodies) to amplify the patient’s own antitumor immune response [7]. Checkpoint inhibitors have previously shown strong efficacy in extracranial advanced melanoma and NSCLC. However, the intracranial immune response is highly regulated and most checkpoint inhibitor clinical trials have excluded patients with BM [82]. More recent studies have yielded promising results regarding the use of checkpoint inhibitors for treatment of melanoma and NSCLC BM, and additional trials are underway (Table 4) [51, 83].

**Table 4:** Selected clinical studies of immune checkpoint inhibitors for brain metastases

Authors & Year	Regimen	Mechanism	Primary cancer	***N***	cRR (%)	PFS (mo)	OS (mo)
Margolin et al., 2012	IPI	CTLA-4	Melanoma	72	10-25	--	3.7-7.0
Di Giacomo et al., 2012 (NIBIT-M1)	IPI + FTM	CTLA-4	Melanoma	20	50	3.0	12.7
Kluger et al., 2019	Pembrolizumab	PD-1/PD-L1	Melanoma	23	26	2	17
Goldberg et al., 2016	Pembrolizumab	PD-1/PD-L1	NSCLC	18	33	--	7.7
Tawbi et al., 2018	Nivolumab + IPI	PD-1/PD-L1 + CTLA-4	Melanoma	94	55	--	--
Long et al., 2018	Nivolumab +/- IPI	PD-1/PD-L1 +/- CTLA-4	Melanoma	76	6-46	2.3-NR	5.1-NR

Abbreviations: cRR: CNS response rate; FTM: fotemustine; IPI: ipilimumab; mo: months; *N*: # patients; NR: not reached; OS: overall survival; PFS: progression free survival.

### Anti-CTLA-4 antibody

Ipilimumab is a monoclonal antibody directed against CTLA-4, which is involved in downregulating cytotoxic T-cell production, and is approved for treatment of metastatic melanoma [84]. Systemic response rates in melanoma patients range from 11-21%, with better response reported in patients with BRAF wild-type melanoma with PD-L1 expression [85, 86]. Intracranial activity of ipilimumab was first described in a post hoc analysis of a phase III trial which included 82 patients with asymptomatic BM and found reduced mortality with the agent [87]. This was followed by an open-label phase II study of ipilimumab that included 72 patients (51 asymptomatic, 21 symptomatic) with melanoma BM [88]. Intracranial response was seen in 13 of 51 (25%) asymptomatic patients and two of 21 (10%) symptomatic patients. Median OS was 7.0 months in asymptomatic and 3.7 months in symptomatic patients. Another open-label phase II trial administered combination ipilimumab-fotemustine to patients with metastatic melanoma with and without CNS involvement (NIBIT-M1) [89]. The study included data on 20 patients with asymptomatic BM who had intracranial response rate of 10 out of 20 (50%), median OS of 12.7 months, and PFS of 3.0 months. Following the optimistic results of the phase II trial, a phase III trial comparing fotemustine versus fotemustine-ipilimumab versus ipilimumab-nivolumab in melanoma patients with BM is currently underway (NIBIT-M2, NCT02460068). In summary, the anti-CTLA-4 antibody ipilimumab has demonstrated efficacy against melanoma BM and additional data are accruing.

### Anti-PD-1/PD-L1 antibodies

Nivolumab and pembrolizumab are anti-PD-1/PD-L1 antibodies which have been approved for advanced melanoma and NSCLC [7]. They have been found to be superior to ipilimumab for advanced melanoma with systemic response rates ranging from 33-57%, but these studies excluded patients with BM [85, 90]. A recently completed single-center, phase II study of pembrolizumab enrolled melanoma and NSCLC patients with newly diagnosed asymptomatic or progressing BM who did not require immediate treatment with steroids. Among the 18 NSCLC patients, intracranial response rate was six (33%, four complete and two partial), and median OS was 7.7 months [91]. In the 23 melanoma patients, intracranial response rate was six (26%, four complete and two partial), OS was 17 months, and PFS was 2 months [92]. Another multicenter phase II trial of ipilimumab-nivolumab included data on 94 patients with advanced melanoma and asymptomatic BM [93]. This study found intracranial response rate of 55% (24 complete, 28 partial responses). Finally, a multicenter, open-label, randomized phase II trial reported on 60 patients with melanoma and asymptomatic BM with no previous local therapy who received ipilimumab-nivolumab (cohort A) or nivolumab alone (cohort B) [94]. The study also included 16 patients with BM who had neurological symptoms, leptomeningeal disease, or local therapy failure and underwent therapy with nivolumab alone (cohort C). Intracranial response rates were 16 of 35 (46%) in cohort A, five of 25 (20%) in cohort B, and one of 16 (6%) in cohort C. Median OS was not yet reached in cohort A, was 18.5 months in B, and was 5.1 months in C. Median intracranial PFS was not reached in cohort A, was 2.5 months in B, and was 2.3 months in C. Overall, the anti-PD-1/PD-L1 antibodies, nivolumab and pembrolizumab, appear to be effective in treating intracranial manifestations of metastatic melanoma; pembrolizumab may also be efficacious in managing NSCLC BM.

### Predicting response to immune checkpoint inhibitors

The identification of biomarkers to predict response to immune checkpoint inhibitors has been explored in recent years. Histopathological studies have demonstrated critical links between tumor-infiltrating immune cell density and distribution, PD-1 expression in immune cells, and PD-L1 expression in tumor cells with overall disease prognosis in patients with lung cancer and melanoma BM [82, 95–97]. For instance, high tumor PD-L1 expression has been widely explored as a potential predictive biomarker for selecting patients who will derive benefit from anti-PD-1/PD-L1 therapy in primary NSCLC and melanoma, but studies have not specifically focused on BM [98, 99]. Furthermore, interferon-γ is an important regulator of immunity that is produced by natural killer and activated T-cells and induces PD-L1 expression as an adaptive response to endogenous antitumor immunity in numerous primary extracranial cancer cells, as well as in multiple components of the glioma microenvironment [100, 101]. Expression of interferon-γ and interferon-γ-inducible genes correlate with response to anti-PD-1/PD-L1 agents in primary melanoma and NSCLC: patients with both interferon-γ and PD-L1-positivity (as defined by greater than 25% of tumor cells) demonstrated the highest response rates [100, 102, 103]. Additionally, high somatic mutational burden as characterized by whole-exome sequencing or various next generation sequencing panels has been correlated with sustained clinical benefit from immune checkpoint inhibitors in a growing number of extracranial cancers including melanoma, NSCLC, urothelial cancer, and head and neck squamous cell carcinoma [104–107]. Both FoundationOne CDx and MSK-IMPACT sequencing panels have been approved by the FDA for measurement of tumor mutational burden [108]. Finally, studies have suggested that activation of the Ras-MAPK pathway may be a useful biomarker for predicting response to immune checkpoint inhibitors [109–111]. In general, although these biomarkers show promise in primary NSCLC and melanoma, additional studies validating their ability to predict response to immunotherapy specifically in intracranial disease are still needed.

## IMPLICATIONS FOR PRACTICE AND FUTURE DIRECTIONS

For targeted agents and immune checkpoint inhibitors, baseline MRI should be performed within a month prior to initiating treatment, followed by an image approximately every two to three months for the first year and an increasing interval thereafter. This interval has not been studied rigorously, but allows early detection of treatment failure and switching to more traditional therapies if indicated.

To assess patients receiving immunotherapy, the immunotherapy Response Assessment for Neuro-Oncology (iRANO) criteria were developed [112]. Importantly, if the contrast-enhanced MRI suggests progression within the first 6 months of treatment, there should be a confirmation image before stopping therapy. The goal of this recommendation is to allow for the possibility that significant enhancement may reflect an inflammatory response to the tumor, and an efficacious response. Waiting for this confirmatory MRI, optimally up to three months later, is dependent on the patient being clinically stable. This is a complicated point, as advancing symptoms may be secondary to a worsening brain lesion, regardless of the etiology being anti-tumor inflammation or tumor progression. Another point is that after treatment with immunotherapy, new enhancing lesions may appear as a result of anti-tumor responses against previously unknown but present lesions that were not detected on MRI. In this case, new changes on an MRI reflect response and not treatment failure. Lastly, if there has been a decrease in steroids within two weeks of MRI, the MRI-detected lesion cannot necessarily be called progressive, but rather non-evaluable. Patients with significant changes by iRANO criteria after 6 months of initiating immunotherapy are considered as no longer deriving benefit.

MRI sequences such as perfusion and cerebral blood volume are used in some centers to try to distinguish between inflammation and tumor progression, but there is little validation data published. PET/MRI is another potentially useful tool. A hypometabolic lesion on PET/MRI increases the confidence that the change is less likely tumor progression; however, there are not yet norms to define how much avidity inflammatory responses may have.

Finally, additional phase 0 trials are needed to test whether targeted systemic therapies are penetrating BM. These trials would involve upfront treatment with targeted agents followed by surgical resection and ideally both determination of drug concentration and evidence of “on-target” activity within resected tissue. Such trials could have the additional benefit of validating surrogate response biomarkers, which would optimally be less invasive than needle biopsy or surgical resection.

## CONCLUSIONS

Local treatment with surgery, SRS, and WBRT remain the mainstays of therapy for BM, but emerging evidence regarding the efficacy of targeted systemic treatments and immunotherapy continues to accumulate. For NSCLC, numerous clinical trials have demonstrated CNS activity for inhibitors of EGFR and ALK. Systemic therapy using BRAF-inhibitors with and without trametinib has resulted in encouraging outcomes for patients with melanoma BM. Several targeted options are available for breast cancer BM that overexpress HER2. Immune checkpoint inhibitors including anti-CTLA-4 and anti-PD-1/PD-L1 antibodies are yielding impressive responses in melanoma and NSCLC BM. Management of eligible patients will require increased multidisciplinary discussion incorporating novel systemic treatment approaches prior or in addition to local therapy. In these cases, close follow-up with CNS imaging is necessary to evaluate response to targeted agents, with surgery or radiation as alternatives at disease progression.
